# A case of severe polyarteritis nodosa with critical limb threatening ischemia—promising treatment with sirolimus drug-coated angioplasty

**DOI:** 10.1016/j.jvscit.2023.101266

**Published:** 2023-07-17

**Authors:** Darius Kang Lie Aw, Kalpana Vijaykumar, Shin Chuen Cheng, Tjun Yip Tang, Jia Sheng Tay, Edward Tieng Chek Choke

**Affiliations:** Department of Vascular Surgery, Seng Kang General Hospital, Singapore

**Keywords:** Critical limb threatening ischemia, Percutaneous transluminal angioplasty, Polyarteritis nodosa, Sirolimus, Vasculitis

## Abstract

Polyarteritis nodosa (PAN) is a rare form of vasculitis. Acute limb ischemia is a rare presentation and complication of PAN. Plain old balloon angioplasty (POBA) is one of the treatment strategies for addressing PAN-related critical limb threatening ischemia (CLTI). However, recurrence of stenosis and occlusion is frequent, making POBA a poor treatment choice, as evidenced in our described clinical case. Consequently, with consideration of sirolimus's anti-inflammatory and immunosuppressive properties, we used a sirolimus-coated balloon in the treatment of PAN-induced CLTI. A 37-year-old woman first presented with acute limb ischemia as her initial symptom. Diagnostic angiography demonstrated occlusion of her tibial vessels, and POBA was performed to restore perfusion. Later in the course of her illness, she developed foot gangrene despite multiple courses of immunosuppressive drugs and several attempts with POBA to achieve limb salvage. Because of her disease trajectory, a MagicTouch (Concept Medical) sirolimus-coated balloon was deployed to her anterior tibial artery during her third angioplasty. At 17 months after her last angioplasty, she remained ulcer free, and surveillance scans demonstrated occlusion-free tibial vessels. The use of sirolimus-coated balloon angioplasty is a promising treatment approach for successful limb salvage in patients with PAN vasculitis and CLTI.

Polyarteritis nodosa (PAN) is a rare form of systemic necrotizing vasculitis in the adult population. It is a systemic disease that typically affects medium-size arteries, with occasional involvement of small muscular arteries.^1^ The pathogenetic mechanisms of PAN are not well understood. Thickening of the inflamed vessel wall and intimal proliferation can cause luminal narrowing and a higher thrombotic risk of the affected vessels.

PAN usually presents with systemic symptoms and has a predilection for the kidneys, nerves, and mesenteric arteries. Consequently, premature peripheral vascular disease (PVD) in young patients is extremely uncommon. Studies characterizing its prevalence, causes, and treatment approaches are lacking. Few studies have reported PAN presenting as PVD^2^^,^^3^ and acute limb ischemia.^4^ Plain old balloon angioplasty (POBA) is one of the treatment choices for below-the-knee arterial stenosis and occlusion, although the recurrence of stenosis and occlusion, as illustrated by our clinical case, is frequent. We hypothesize that this is likely due to the continuous intimal proliferation and inflammation in PAN, which is made worse by a viscous cycle resulting from the use of POBA with accelerated migration of cytokines and neointimal proliferation. Hence, we decided to apply endoluminal local anti-inflammatory and immunosuppressive effects on the targeted vasculature in the hope of arresting the vasculitis process that occurs with PAN.

We describe a case in which sirolimus-coated angioplasty was performed on the anterior tibial artery (ATA), achieving primary patency with successful limb salvage in a patient with PAN with CLTI.

## Case report

A 37-year-old woman presented to the emergency department in October 2020 with acute left leg pain of 4 hours’ duration. On clinical examination, the foot was pale and cold, with associated numbness. Her heart rate was regular, and electrocardiography did not reveal evidence of atrial fibrillation. Acute limb ischemia was diagnosed. Analgesia and a bolus heparin dose of 5000 U were administered. She was immediately brought to the operating theater. A 5F sheath (Terumo Corp) was inserted antegrade in the left common femoral artery. Diagnostic angiography demonstrated patent above-the-knee and proximal tibial arteries. However, total occlusion of the distal below-the-knee arteries was present (Fig). Rheolytic thrombolysis and thrombectomy with the AngioJet thrombectomy system (Boston Scientific) and POBA were performed to the ATA and posterior tibial artery (PTA), with reestablishment of an intact pedal plantar arch to the foot (Fig). Intraoperatively, severe vasospasm was observed, and 1000 μg of nitroglycerine was administered to restore luminal patency. Angiography revealed dilated areas alternating with narrowing of the blood vessels; “beading” was also evident during surgery (Fig). At the end of her first angioplasty, her foot perfusion improved and color returned. Both dorsalis pedis and PTA pulses were palpable at end of the procedure.

**Fig:**
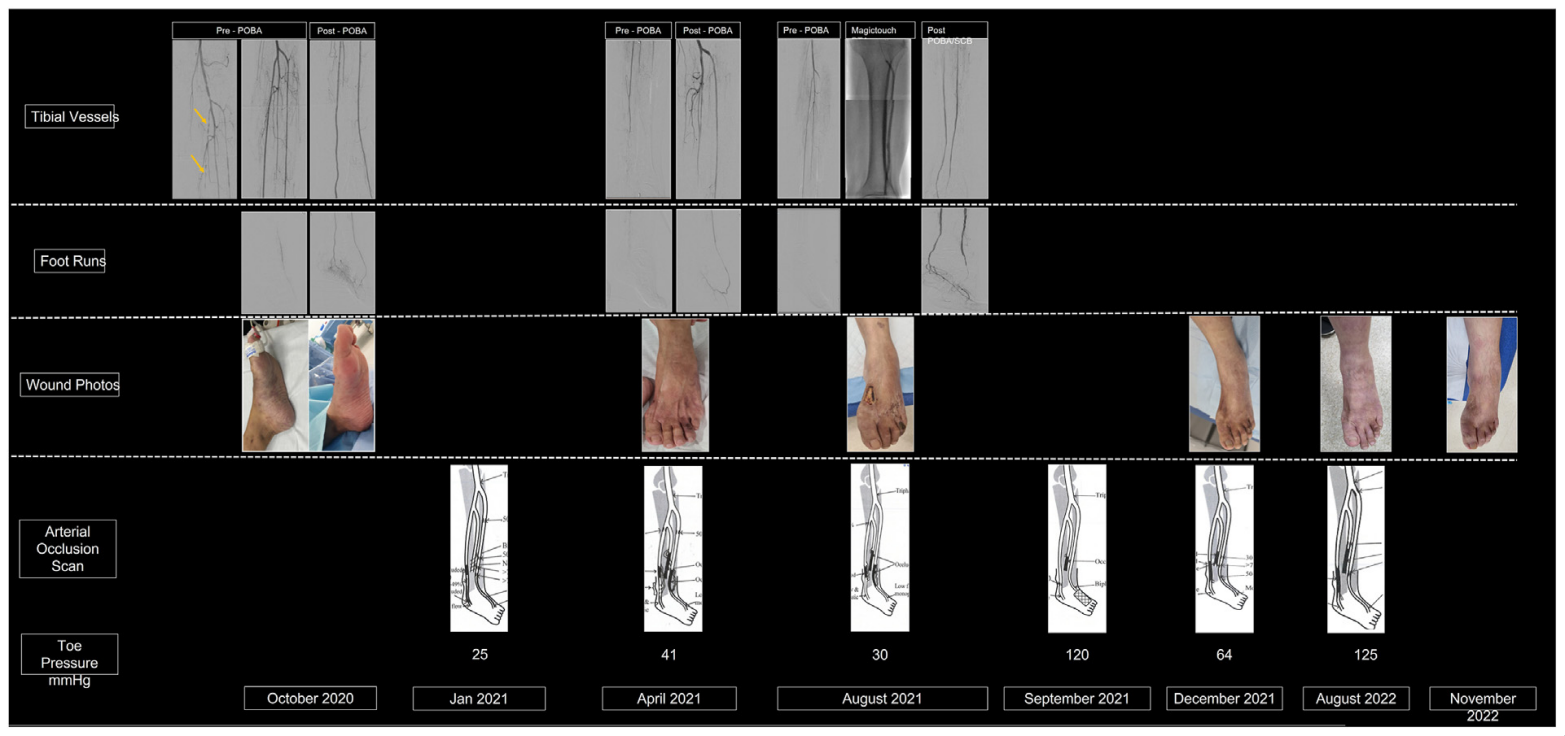
Timeline of patient with polyarteritis nodosa (PAN)-induced critical limb threatening ischemia (CLTI). She first presented with left acute limb ischemia with a mottled dusky foot and required emergent angioplasty to restore perfusion. Intraoperatively, angiographic evidence of “beading” with alternating dilatation and narrowing was observed (*yellow arrow*). Sequential angiography also revealed disease progression, with worsening distal tibial vessel patency and the last angiography demonstrating a “desert foot.” During her disease, she continued to deteriorate and experienced CLTI with tissue loss, requiring repeated angioplasty over a span of 10 months. In her third angioplasty, we used the MagicTouch, a sirolimus-coated percutaneous transluminal angioplasty catheter, on the anterior tibial artery (ATA), in the hope of arresting the vasculitis progression with the anti-inflammatory and immunosuppressive properties of sirolimus. Her foot healed within 4 months after sirolimus treatment, and she remained intervention free at 17 months and ulcer free at 13 months after her last treatment. Surveillance arterial occlusion ultrasound scans at 1, 6 and 12 months demonstrate that her ATA remained patent with no occlusion. She continued to have a strong dorsalis pedis pulse at her last follow-up at 17 months.

At the postoperative examination, she reported recurrent symptoms experienced before her acute presentation, suggestive of Raynaud phenomenon. Autoimmune blood markers were tested, and the test for perinuclear antineutrophil cytoplasmic antibodies was positive. Before her repeat presentation to the vascular team a few months postoperatively, her symptoms of pain and numbness progressed. She experienced vasculitic rash and livedo reticularis over her extremities and mononeuritis multiplex over her foot. Biopsies were performed, and histologic examination demonstrated perivascular inflammatory infiltrates and vasculitis. With the clinical manifestations and biochemical and histologic evidence, PAN was diagnosed. Intravenous steroids and oral methotrexate were started, which required escalation to intravenous cyclophosphamide, with some relief.

During the course of her treatment 5 months after her first angioplasty, she started to develop a superficial ulcer over the dorsum of the fourth toe. Arterial duplex ultrasound revealed reocclusion of her distal tibial vessels. With evidence of tissue loss, she returned for her second angioplasty. The PTA was chronically occluded with unsuccessful crossing, and her ATA had acute on chronic occlusion with thrombus. The ATA was successfully treated with the AngioJet systems and POBA, with restoration of blood flow into the left foot. She was given dual antiplatelet therapy and a novel oral anticoagulant (rivaroxaban 5 mg twice daily) after surgery.

However, avoidance of early tibial vessel reocclusion and avoidance of new tissue loss were not achieved after her second angioplasty. She was scheduled for her 3-month regular clinical assessment and duplex ultrasound surveillance scan. However, she did not return for that follow-up visit. She had started to develop a new wound over the dorsum of her foot with worsening foot pain when she presented again to the team 4 months after her second angioplasty.

Arterial duplex ultrasound demonstrated similar occlusion again in the distal tibial arteries. She returned for her third angioplasty. Intraoperatively, POBA to her PTA and ATA was successful with good flow into her foot. However, given the trajectory of her disease progression and dismal systemic control with immunosuppressive medication, we decided to access the anti-inflammatory and immunosuppressive properties of sirolimus. Thus, we decided to treat her ATA with the MagicTouch (Concept Medical) sirolimus-coated balloon at 12 atm and a total inflation time of 2 minutes. Sirolimus-coated balloon angioplasty was only performed for the ATA, with the dorsalis pedis treated only with POBA. She also started biologic therapy with intravenous rituximab after her third angioplasty.

However, despite the rituximab, she continued to have active vasculitis 1 month after her third angioplasty, resulting in a ruptured pseudoaneurysm of her medial lenticulostriate artery with subarachnoid hemorrhage and intraventricular extension, needing coil embolization. She also continued to develop new symptoms of ocular mononeuritis multiplex. Even during biologic therapy, she subsequently experienced another active vasculitis flare 8 months after her third and last angioplasty, in which antitumor necrosis factor was initiated.

Despite the active phase of vasculitis in the 8 months after her third angioplasty with the sirolimus-coated balloon, her foot wound had healed completely at 4 months after that angioplasty. This led us to believe that it was more likely the local effects from sirolimus-coated angioplasty than the systemic treatment that had led to her wound healing. Surveillance arterial duplex ultrasound of her ATA revealed that the ATA remained occlusion free with biphasic blood flow and reasonable toe pressure with a range of ∼97 to 98 mm Hg at 9 months after her third angioplasty. She continued to have a palpable dorsalis pedis pulse with no tissue loss at her last follow-up in January 2023. She has remained intervention free for 17 months and ulcer free for 13 months after the use of sirolimus-coated balloon angioplasty for her vasculitis-induced CLTI. She will continue her follow-up with the vascular team every 6 months or sooner if new symptoms warrant an earlier review.

Given such encouraging and promising results, the patient provided written informed consent for the report of her clinical case details and imaging studies.

## Discussion

PVD is common in the older general population. It usually progresses over time and is associated with risk factors such as smoking and diabetes mellitus. PVD commonly presents with chronic claudication of the lower limbs. However, if PVD appears in a younger patient or an older patient in the absence of traditional risk factors for atherosclerosis, other causes must be considered. These conditions include vasculitis, fibromuscular dysplasia, and popliteal artery entrapment syndrome.

PAN is systemic vasculitis with a low prevalence of ∼6 per 100,000 persons.^5^ Histologically, it is characterized by focal panmural necrotizing arteries affecting small and medium-size arteries.^6^ Because no specific diagnostic investigations are available for PAN, the diagnosis is determined by abnormal angiographic findings and biopsy findings of fibrinoid necrosis with polymorphonuclear infiltrate suggestive of small to medium vessel vasculitis.^7^ Despite the systemic distribution of the arterial lesions, reports of tibial vessel involvement resulting in CLTI are rare.

Several cases of PAN among adults have been reported,^4^^,^^8^ with adults presenting with rapidly progressive intermittent claudication. These patients did not warrant emergent surgical intervention and were treated medically over a period with favorable outcome. However, our patient presented acutely with ischemia with a threatened foot and rapidly progressed to CLTI, with tissue loss despite aggressive medical therapy. She had recurrent flares, even with biologic therapy, with active episodes of vasculitis. This was challenging, because it showed that her disease condition was not well controlled. Repeated angioplasty in an attempt to salvage her foot was performed with good results at each round but recurrence with stenosis and occlusion continued to hamper her recovery. It was the third angioplasty in which we used the sirolimus-coated balloon that was the turning point of her limb salvage journey. We speculated that the anti-inflammatory and immunosuppressive properties of sirolimus could have provided local effects by retarding the arterial wall inflammatory infiltrate and the proliferation of the cells that leads to thickening of the wall and progressive narrowing of the lumen. At her last follow-up, she remained ulcer free with a clinically palpable pulse.

## Conclusions

PAN can be a life- and limb-threatening vasculitis that can uncommonly present with rapid progression. Early diagnosis and angiography are useful. PTA in patients who present with CLTI in the setting of PAN is often a temporizing measure, even with good systemic therapy. The use of sirolimus-coated angioplasty could provide a durable solution to the recurrent stenosis and occlusion induced by PAN and, possibly, successful limb salvage.
